# SECRET AGENT O-GlcNAcylates Hundreds of Proteins Involved in Diverse Cellular Processes in Arabidopsis

**DOI:** 10.1016/j.mcpro.2024.100732

**Published:** 2024-02-08

**Authors:** Ruben Shrestha, Sumudu Karunadasa, TaraBryn S. Grismer, Andres V. Reyes, Shou-Ling Xu

**Affiliations:** 1 Department of Plant Biology, Carnegie Institution for Science, Stanford, California, USA; 2 Carnegie Mass Spectrometry Facility, Carnegie Institution for Science, Stanford, California, USA

**Keywords:** O-GlcNAcylation, mass spectrometry, lectin-weak affinity chromatography, SILIA, PRM

## Abstract

O-GlcNAcylation is a critical post-translational modification of proteins observed in both plants and animals and plays a key role in growth and development. While considerable knowledge exists about over 3000 substrates in animals, our understanding of this modification in plants remains limited. Unlike animals, plants possess two putative homologs: SECRET AGENT (SEC) and SPINDLY, with SPINDLY also exhibiting O-fucosylation activity. To investigate the role of SEC as a major O-GlcNAc transferase in plants, we utilized lectin-weak affinity chromatography enrichment and stable isotope labeling in Arabidopsis labeling, quantifying at both MS1 and MS2 levels. Our findings reveal a significant reduction in O-GlcNAc levels in the *sec* mutant, indicating the critical role of SEC in mediating O-GlcNAcylation. Through a comprehensive approach, combining higher-energy collision dissociation and electron-transfer high-energy collision dissociation fragmentation with substantial fractionations, we expanded our GlcNAc profiling, identifying 436 O-GlcNAc targets, including 227 new targets. The targets span diverse cellular processes, suggesting broad regulatory functions of O-GlcNAcylation. The expanded targets also enabled exploration of crosstalk between O-GlcNAcylation and O-fucosylation. We also examined electron-transfer high-energy collision dissociation fragmentation for site assignment. This report advances our understanding of O-GlcNAcylation in plants, facilitating further research in this field.

## Highlights

• SILIA/PRM reveals the prominent role of SEC in O-GlcNAcylation in Arabidopsis.
• Remarkable expansion of O-GlcNAc substrates with the addition of 227 new targets.
• O-GlcNAc found in similar protein family regions without consensus.
• One-third targets overlap with O-fucosylation targets.
• The study of EThcD underscores the need for ongoing algorithm optimization.

## Graphical Abstract

**Figure:**
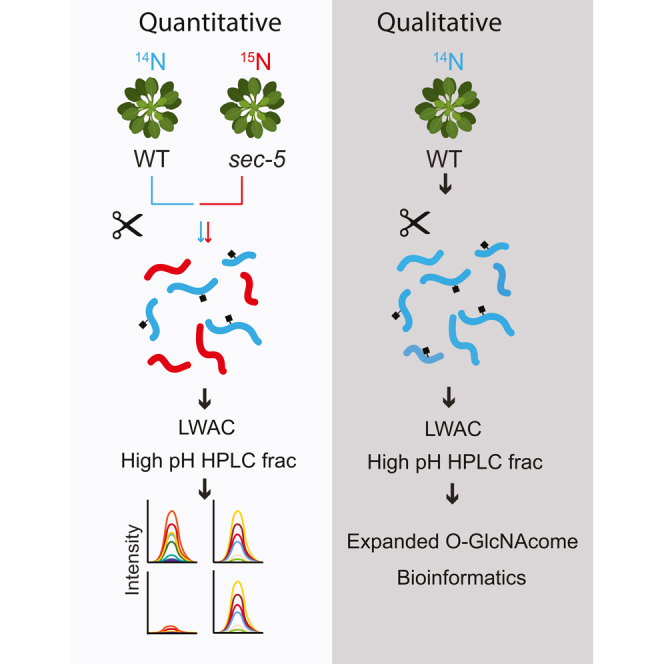

## In Brief

O-GlcNAcylation, crucial for plants but poorly understood, was explored using LWAC and SILIA–PRM quantitative proteomics. We unveiled the prominent roles of SEC in O-GlcNAcylation. Through extensive fractionations, we expanded the O-GlcNAcome by adding 227 new targets, revealing broad regulatory roles and overlapping role with O-fucosylation.

Protein O-GlcNAcylation is a unique post-translational modification (PTM) involving the addition of a single monosaccharide, GlcNAc, to the hydroxyl group of serine residues or threonine residues on nuclear, cytoplasmic, or mitochondrial proteins. This modification, discovered in both animals and plants, has been shown to play an essential role in growth and development (1, 2). O-GlcNAcylation has been demonstrated to participate in a diverse range of cellular processes, including signal transduction, transcriptional regulation, stress response, cellular differentiation, and protein degradation. Dysregulation of this PTM has been linked to various diseases, including Alzheimer’s disease, cardiovascular disease, diabetes, and cancer (3).

O-GlcNAcylation is catalyzed by O-GlcNAc transferase (OGT), comprising an N-terminal tetratricopeptide repeat domain for protein interactions and a C-terminal catalytic domain. OGT acts as a nutrient sensor (4), responding to hexosamine biosynthetic pathway flux and UDP-GlcNAc levels (5). Complete OGT knockout is embryonically lethal in various animal models, whereas knockdown or conditional knockout reveals diverse phenotypic functions. Human OGT (hOGT) exhibits catalytic activity *in vivo* and *in vitro*, being the sole enzyme responsible for this PTM.

In contrast to hOGT, plants have two closely related enzymes, SECRET AGENT (SEC) and SPINDLY (SPY). Knocking out of SPY leads to mild effects, including a spindly shoot, reduced gibberellin sensitivity, male sterility, early flowering, and altered phyllotaxy (6). *sec* mutants are minimally affected, except for some impact on virus infection efficiency (7) and early flowering (8). Double mutants of SEC and SPY are lethal (9, 10). *In vitro*, SEC has shown O-GlcNAc transferase activity using *Escherichia coli* (10), whereas SPY lacks such activity. However, SPY exhibits O-fucosylation activity both *in vitro* and *in vivo* (11). Extensive quantification studies comparing WT and *spy* mutants, after enriching O-fucosylated peptides, highlight SPY as the primary enzyme responsible for O-fucosylation activity (12, 13).

Recently, a discovery in *Triticum aestivum* (bread wheat) revealed an atypical TaOGT (TaOGT1) with no structural similarity to SEC and SPY enzymes (14). TaOGT1 was found to O-GlcNAcylate TaGRP2. In Arabidopsis, approximately 34 unannotated or uncharacterized proteins share similarity with TaOGT1, leading to questions about whether SEC is the primary contributor to O-GlcNAcylation in plants.

Comparatively, although over 3000 and 1430 O-GlcNAc targets have been identified in humans and mice, respectively (15, 16), the database for O-GlcNAc targets in plants remains limited (17, 18, 19, 20). In addition, given the overlapping or even opposite functions of O-GlcNAcylation and O-fucosylation, identifying more common targets would help in understanding their roles. Previously, we identified 262 O-GlcNAc targets (20) and 467 O-fucosylated targets in Arabidopsis (12).

In this study, we conducted genetic, quantitative proteomic, and large-scale proteomic experiments to investigate O-GlcNAcylation. We confirmed that SEC and SPY are essential for embryo cell viability. Through quantitative proteomics, we demonstrated that SEC is a major contributor to O-GlcNAcylation in plants. Moreover, we significantly expanded the O-GlcNAc target identifications, discovering a total of 436 O-GlcNAc targets, including 227 new targets. In addition, we explored the crosstalk between O-GlcNAcylation and O-fucosylation. Finally, we delved into the complexity of site assignment using electron-transfer high-energy collision dissociation (EThcD) for O-GlcNAc studies.

## Experimental Procedures

### Plant Growth and Tissue

WT (Col), *sec-5* (SALK_034290) and *spy-3*, and *sec-5* (−/−) *spy-3*(+/−) were grown in the greenhouse for 30 to 40 days. Siliques were taken and examined under the Amscope MD500 microscope. Genotyping primers (*sec-5* genotyping primers—JMLB1-GGCAATCAGCTGTTGCCCGTCTCACTGGTG, SEC RF1-tcccgacctgtctttctttccgat, SEC RR1-ttccacgcgtttgccatgtttc, *spy-3* genotyping primers—JP91-GCGACCTATCACCATTGGA, SPY LPD3-TCGACCTGCCTGCAATCAAA).

### Stable Isotope Labeling in Arabidopsis

The WT (Col) and *sec* seedlings were grown on Hoagland medium containing ^14^N or ^15^N isotope salt (1.34 g/l Hoagland's No. 2 salt mixture without nitrogen [Caisson Labs], 6 g/l Phytoblend, and 1 g/l KNO_3_ or 1 g/l K^15^NO3 [Cambridge Isotope Laboratories], pH 5.8) for 14 days. Plates were placed vertically in a growth chamber under constant light conditions at 22 °C. Similarly, a single biological replicate of Col and *spy-4* was labeled by ^14^N and ^15^N isotopes, respectively, for subsequent large-scale identification of O-GlcNAc peptide.

### Stable Isotope Labeling in Arabidopsis Sample Preparation

Whole plant tissues were harvested and cryomilled in liquid nitrogen. For quantifications, the tissues from six samples were equally mixed to make three biological replicates for both WT and *sec-5* mutant (F1: ^14^N Col/^15^N *sec-5*; F2: ^14^N Col/^15^N *sec-5*; and R1: ^15^N Col/^14^N *sec-5*). Similarly, one replicate was prepared for WT and *spy-4* mutants (S1: ^14^N Col/^15^N *spy*-*4*). Protein extraction was performed using phenol extraction method as described (20). Following protein extraction, proteins were reduced and alkylated followed by trypsin digestion. Peptides were desalted using Sep-PAK C18 cartridges (Waters).

### Experimental Design and Rationale

For quantitative analysis of O-GlcNAc levels between WT and *sec-5* mutant, stable isotope labeling in Arabidopsis (SILIA) was chosen for its amino acid–level labeling and early sample mixing, minimizing technical variabilities. These experiments, typically conducted in two replicates, involve switching the labeling in second replicate (21, 22, 23). Targeted quantification using parallel reaction monitoring (PRM) (24, 25) was done on the F2, R1 samples after data-dependent acquisition (DDA) on F2 replicates. The DDA provided necessary retention time/fractionation numbers. Importantly, the MS2 of these light and heavy pairs produces the same fragments with different mass, but with the same pattern (the order of the most abundant to the least fragments is the same), resulting in minimal false discovery rates for these peptides. ^15^N-labeled peptides serve the same role as “natural” heavy peptides, adding an extra layer of reliability. Additional DDA quantification (MS1) data in F1, F2, on a list of preselected modified peptides with detected sugar neutral loss in MS2, were included, offering an extra layer of quantification at the MS1 level.

For qualitative identification of O-GlcNAc peptides, all DDA runs (a total of 107 runs) from samples prepared in the “*SILIA labeling*” and “Lectin-weak affinity chromatography enrichment and high-pH reverse fractionation” sections were included.

### Lectin-Weak Affinity Chromatography Enrichment and High-pH Reverse Fractionation

The lectin-weak affinity chromatography (LWAC) column was packed as described (20), with slight modification for three-round glycosylated peptide enrichment. Peptides were resuspended in 100 μl LWAC buffer (100 mM Tris, pH 7.5, 150 mM NaCl, 2 mM MgCl_2_, 2 mM CaCl_2_, 5% [v/v] acetonitrile). Chromatography was performed at a flow rate of 100 μl/min, with a single glycopeptide-enriched tail collected after 1.3 ml.

To prevent column overload, in the initial LWAC enrichment, total peptides were divided into aliquots of ∼1 to 2 mg each and run separately. In Col/*sec-5* experiments, the starting peptides were as follows: F1: 11 mg (split into six runs with 1.8 mg injection each), F2: 5 mg (split into five runs with 1 mg injection each), and R1: 5 mg (split into five runs with 1 mg injection each). For S1 Col/*spy-4* experiment, the starting peptides were 20 mg (split into 10 runs with 2 mg injection each).The glycopeptide-enriched fractions were combined and desalted for the subsequent round. Subsequent rounds utilized peptides from the prior round, with the same glycopeptide-enriched fraction being targeted. Column cleaning involved injecting 100 μl of 40 mm GlcNAc in the LWAC buffer to elute any bound glycopeptides.

The high-pH reverse HPLC fractionations were performed as described (20) and collected as described in supplemental Data S8. For F1: ^14^N Col/^15^N *sec-5*, eight fractions were collected. For F2: ^14^N Col/^15^N *sec-5*, 18 fractions were collected for DDA assay, and 10 of these underwent targeted assay. For R1: ^15^N Col/^14^N *sec-5*, 10 fractions were collected and subjected to targeted assay. For S1: ^14^N Col/^15^N *spy-4*, 52 extensive fractions were collected and analyzed through DDA analysis for qualitative identifications of O-GlcNAc-modified peptides.

### Nanoflow LC–MS/MS

Peptides were analyzed by LC–MS/MS on an Easy LC 1200 UPLC liquid chromatography system connected to Q-Exactive HF hybrid quadrupole-Orbitrap mass spectrometer or Orbitrap Eclipse with electron-transfer dissociation (ETD) (Thermo Fisher) (supplemental Data S7). Peptides were separated using Easy-Spray C18 columns (75 μm × 50 cm) (Thermo; ES803A or ES903) or IonOpticks column Aurora UHPLC Emitter column with nanoZero (AUR2-25075C18A), with the exception for F2/R2 datasets, which utilized a trapping column before separation (NanoViper trap column Acclaim pepMap C18 column [75 μm × 2 cm; ThermoFisher, catalog no.: 164946]). The flow rate was 300 nl/min, and a 120 min gradient was employed. Peptides were eluted by a gradient from 3 to 28% solvent B (80% [v/v] acetonitrile/0.1% [v/v] formic acid) over 100 min, followed by an increase to 44% solvent B over 20 min, and concluded with by a short wash at 90% solvent B.

For data acquired on Q-Exactive HF with DDA, precursor scan was from mass-to-charge ratio (*m/z*) 375 to 1600 (resolution 120,000; automatic gain control [AGC] 3.0E6, maximum injection time 100 ms), and top 20 most intense multiply charged precursors were selected for fragmentation (resolution 15,000, AGC 5E4, maximum injection time 60 ms, isolation window 1.0 *m/z*, scan range 200–2000, spectrum data type profile, minimum AGC target 1.2e3, intensity threshold 2.0 e4, include charge state = 2–8). Peptides were fragmented with higher-energy collision dissociation (HCD) with normalized collision energy (NCE) 27.

For data acquired on Q-Exactive HF with PRM acquisition, data were acquired in the PRM mode. Peptides were similarly separated as described previously. The samples were analyzed using the PRM mode with an isolation window of 1.4 Th. PRM scans were done using 60,000 resolution (AGC target 2e5, 200 ms maximum injection time) triggered by an inclusion list. NCE 27 was used in HCD mode. The PRM data were analyzed using the Skyline software (21, 25). The Skyline templates were generated as described (25) including the light and heavy pairs, and raw data were imported as centroid MS2, allowing a 5 ppm mass error. To select transitions, we chose y-type ion fragments because of their higher ion abundance compared with b-type ion fragments (22). Peaks were manually curated based on the retention window. Contaminating transition peaks that do not coelute were removed. ^14^N peptides and ^15^N peptides should exhibit a similar pattern from the two biological replicate samples (the order of the most abundant to the least abundant MS2 ions should be the same). We quantified 10 peptides with four to five transitions, whereas all the rest of the peptides used six or more transitions to quantify the abundance. Tier 3 level of the targeted MS analysis was applied in the study. Raw data, as well as Skyline data analysis, were included in the PRIDE submission. PXD044831.

For HCD–EThcD data acquired on an Orbitrap Eclipse (Thermo Scientific), the mass spectrometer was coupled with an Easy LC 1200 UPLC liquid chromatography system (ThermoFisher). Peptides were separated using LC gradient, the same described as aforementioned. The precursor ions were scanned from 375 to 1600 *m/z* (resolution 120,000; AGC 4.0E5), and the charge state 2^+^ to 6^+^ was filtered in the quadrupole with a selection window of 1.0 *m/z*, and MIPS peptide filter was enabled. Peptides were fragmented with higher energy collision dissociation (HCD) with NCE 27. Dynamic exclusion was enabled for 10 s. For triggered EThcD, mass trigger was set as 204.0866 with mass tolerance of 25 ppm. Ions were isolated in quadrupole, EThcD with activated (resolution 30,000, AGC target 5E4, normalized AGC target 100%, microscans = 3, with supplemental collision energy 35).

### Data Analysis (SILIA)

MS/MS data were processed using PAVA script, which generates centroid MS2 peaklist. The data analysis was analyzed as described (23) but reports the quantification in peptide level. For data acquired on Eclipse, the peaklists were split into HCD and EThcD peaklist categories. Data were searched using Protein Prospector (version 6.2.1) against the TAIR database *Arabidopsis thaliana* download (https://www.arabidopsis.org/), concatenated with sequence randomized versions of each protein (a total of 35,386 entries). A precursor mass tolerance was set to 10 ppm, and MS/MS2 tolerance was set to 20 ppm. Carbamidomethylcysteine was specified as a constant modification, whereas the variable modifications include protein N-terminal acetylation, peptide N-terminal Gln conversion to pyroglutamate, Met oxidation. In addition, for GlcNAc modifications, HCD-m or EThcD-m1 allows both retention and loss of the modification by searching HexNAc modification on serine, threonine, and asparagine (S/T/N) in the peptides and neutral loss the same time. HCD-m considers b and y ions, whereas EThcD-m1 considers c, z, b, and y ions. In contrast, EThcD-m2, which considers c, z, b, and y ions, but focuses on the retention of the modification by searching for HexNAc modification on S/T/N. The false discovery rate was set at 1% for both proteins and peptides. About 204 filtered lists from HCD peaklists were used for search, alongside the complete peaklists. The cleavage specificity was set to trypsin, allowing up to two missed cleavages and a maximum of three modifications. PRM data quantification was described (25).

### Bioinformatic Analysis

Subcellular localization analysis was performed with SUBA5.0 (https://suba.live/) with default settings (26). The consensus subcellular localization was used. Gene Ontology (GO) analysis was performed using the “Database for Annotation, Visualization and Integrated Discovery” (DAVID) application (DAVID Functional Annotation Bioinformatics Microarray Analysis) (27) with the *A. thaliana* fully annotated protein library as the background.

## Results

### Embryo Lethality of Knocking Down Both *sec* and *spy*

**Fig. 1:**
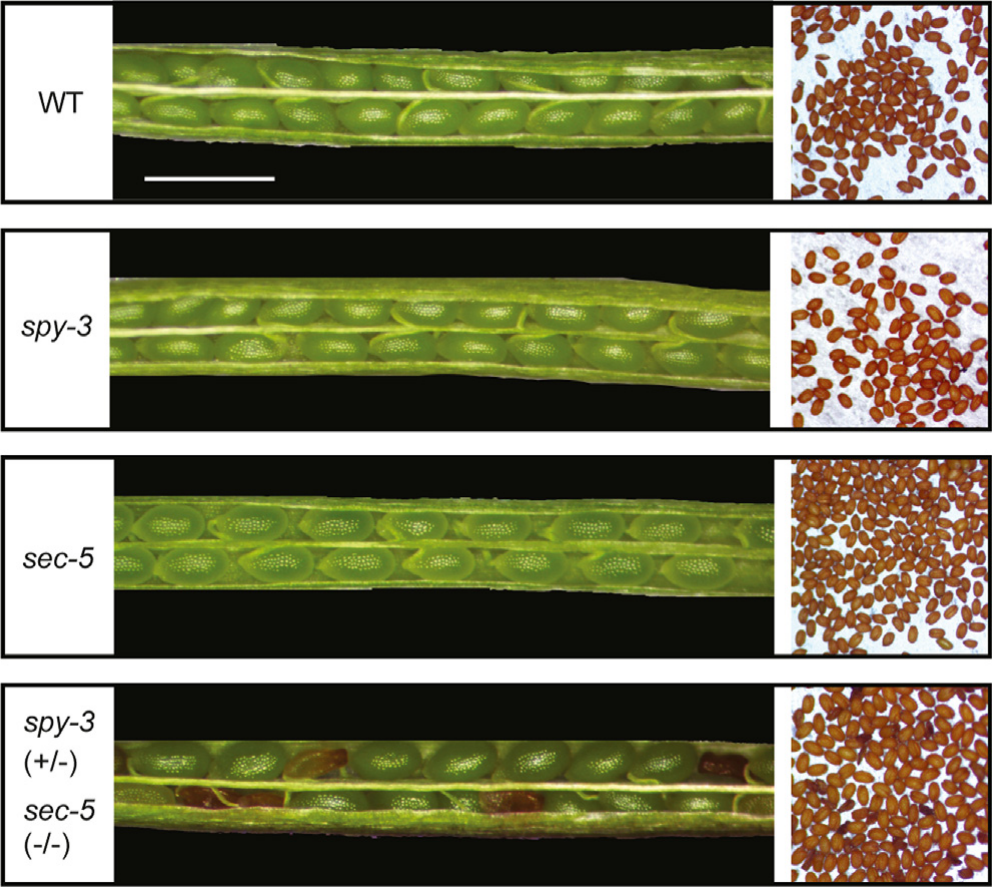
**Essential functions of SEC and SPY in embryo development**. Representative siliques from WT, *spy-3*, *sec-5*, *sec-5(−/−) spy-3(+/−)* plants are shown on the *left*, and seed set for each genotype is shown on the *right*. Siliques from *sec-5*(−/−) *spy-3*(+/−) plants produce approximately 17 to 20% of embryos exhibiting browning and reduced size, which persist throughput maturity. Scale bar represents 1 mm. SEC, SECRET AGENT; SILIA, stable isotope labeling in Arabidopsis; SPY, SPINDLY; S/T/N, serine, threonine, and asparagine.

Previous genetic studies have shown that SEC and SPY have redundant functions during gametogenesis and embryogenesis, despite *sec* single mutants not displaying any severe phenotype (10). This was demonstrated by constructing double mutants using the *spy-3* (point mutation in the catalytic domain, AT3G11540) and *sec-1* or *sec-2* (AT3G04240) allele and not identifying the double mutant alleles in the F2 generation. To further investigate this functional redundancy, we attempted to construct a *sec spy* double mutant by crossing *spy-3* with *sec-5* (8). This *sec-5* allele carries an insertion in the second exon of the SEC genome, resulting in a significant decrease in SEC transcript levels and is considered the strongest allele. As SEC and SPY are genetically linked because of being close on the same chromosome, we screened 520 seeds collected from *sec-5*(−/−) *spy-3*(+/−) plants for germination on paclobutrazol-containing plates and expected to get ¼ double mutant seeds, which would be paclobutrazol resistant. However, we failed to recover any double mutants (data not shown). We also grew these seeds on ½ MS plates and genotyped plants but failed to identify any double mutants (data not shown). We next examined the siliques and observed that while *spy-3* and *sec-5* individually exhibited similar embryos to the WT, siliques from *sec-5*(−/−) *spy-3*(+/−) produced 17 to 20% of embryos that turned brown and shrank to a smaller size (Fig. 1). In addition, we observed shrunken seeds in the mature stage (Fig. 1) that failed to germinate (data not shown). These findings confirm that SEC and SPY have overlapping functions in embryo development and highlight the importance of O-glycosylation, including both O-GlcNAcylation and O-fucosylation, for embryo cell viability.

### Quantifying O-GlcNAc Abundance Levels Between WT *versus sec-5* Mutant

**Fig. 2:**
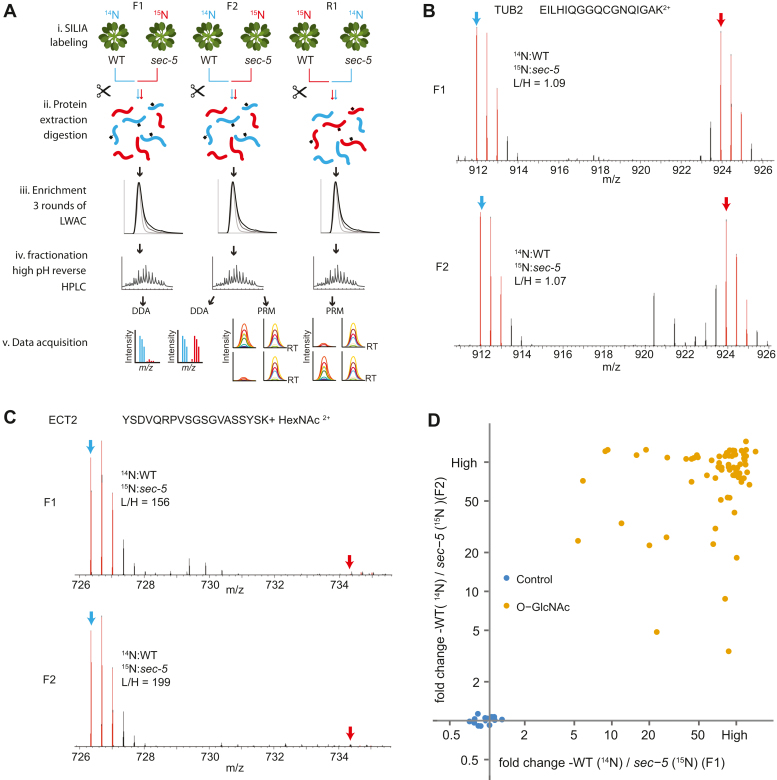
**Quantitative workflow to determine the role of SEC in O-GlcNAcylation in Arabidopsis**. *A*, workflow for stable isotope labeling, LWAC enrichment, high-pH reverse HPLC fractionations, and mass spectrometry (MS) analysis. Total proteins from ^14^N- and ^15^N-labeled WT plants and *sec-5* mutants were mixed, extracted, and digested with trypsin. For each mixture, O-GlcNAc-modified peptides were captured through three rounds of LWAC enrichment and separated by high-pH reverse HPLC fractionations. LC–MS/MS analysis was performed using data-dependent acquisition (DDA) and parallel reaction monitoring (PRM). *B* and *C*, selected MS1 spectra illustrating the relative abundance of nonmodified peptides and O-GlcNAc-modified peptides in WT compared with *sec-5* mutant (F1 and F2 replicates). Spectra represent peptides from TUBULIN BETA CHAIN2 (TUB2, AT5G62690) (*B*) and EVOLUTIONARILY CONSERVED C-TERMINAL REGION 2 (ECT2) (*C*). *D*, scatterplot showing ratios of O-GlcNAc-modified peptides from modified proteins and control peptides from housekeeping proteins. Quantification using SILIA-MS1 was performed in both F1 and F2 replicates comparing WT *versus sec-5* mutants. LWAC, lectin-weak affinity chromatography; SEC, SECRET AGENT.

Next, we determined if SEC is the major contributor to O-GlcNAcylation. We conducted a comparison of O-GlcNAc-modified peptide levels between WT and *sec* mutant samples. To enrich for O-GlcNAc-modified peptides, we utilized the LWAC pipeline as described in our previous study (20). We then quantified the levels of O-GlcNAc between WT and *sec-5* mutant using a workflow involving SILIA and quantified using both DDA and targeted quantification *via* PRM (23, 25) (Fig. 2*A*). SILIA, like stable isotope labeling by amino acids in cell culture, was used in animal cell lines, labels at the amino acid level, and enables early sample mixing, significantly reducing the introduction of technical variabilities in the process. We reasoned that SILIA would be best for our experiments, as the LWAC followed by HPLC fractionations involves many steps and is labor intensive. Typically, these experiments are conducted in two replicates, with the labeling in the second replicate being switched (28, 29, 30).

Briefly, we grew WT and *sec-5* mutant seedlings on media containing the ^14^N and ^15^N stable isotopes (F1 & F2 biological replicates, ^14^N WT and ^15^N *sec-5*) and harvested the plants on day 14. In the third biological replicate (R1), we reversed the isotope labeling (R1, ^15^N WT and ^14^N *sec-5*) (Fig. 2*A*). After protein extraction from mixed tissues and tryptic digestion, each peptide sample was enriched for GlcNAc-modified peptides using three rounds of LWAC and subjected to high pH HPLC fractionation. All peptide samples were analyzed by liquid chromatography coupled with high-resolution and high-accuracy Orbitrap Q-Exactive HF or Orbitrap Eclipse (LC–MS/MS). We observed a significant reduction in the identification of sugar-modified peptides in the ^15^N-labeled WT samples because of incomplete labeling, potentially resulting in problematic monoisotopic precursor assignment in the peaklist generation process with the presence of satellite peaks (such as M-1, M-2 peaks) (Shrestha, unpublished observation) (23, 25). As a result, the F1 and F2 replicates were analyzed using DDA and quantified at the MS1 level, whereas F2 and R1 were quantified using PRM at the MS2 level (Fig. 2*A*).

The SILIA DDA data from F1 and F2 give high-confidence quantification data. Quantified sugar-modified peptides were selected with a 204 neutral loss. The equal mixture of ^14^N-labeled WT and ^15^N-labeled *sec-5* was exemplified in TUBULIN2 quantification (F1, L/H =1.09; F2, L/H = 1.07) (Fig. 2*B*). However, the O-GlcNAc-modified peptide from EVOLUTIONARILY CONSERVED C-TERMINAL REGION2 (ECT2) (Fig. 2*C*) showed a significant reduction in the *sec-5* mutant compared with WT (F1, L/H = 156; F2, L/H = 199). In total, we quantified 72 O-GlcNAc peptides from 57 proteins detected in both replicates (Fig. 2*D* and supplemental Data S1). Apart from TUB2, 12 other housekeeping proteins also verified the equal mixture between WT and *sec-5* (Fig. 2*D*). In contrast, the scatter plot demonstrated that all quantified O-GlcNAc-modified peptides detected in both replicates consistently exhibited a significant reduction in the *sec-5* mutant (Fig. 2*D*).

**Fig. 3:**
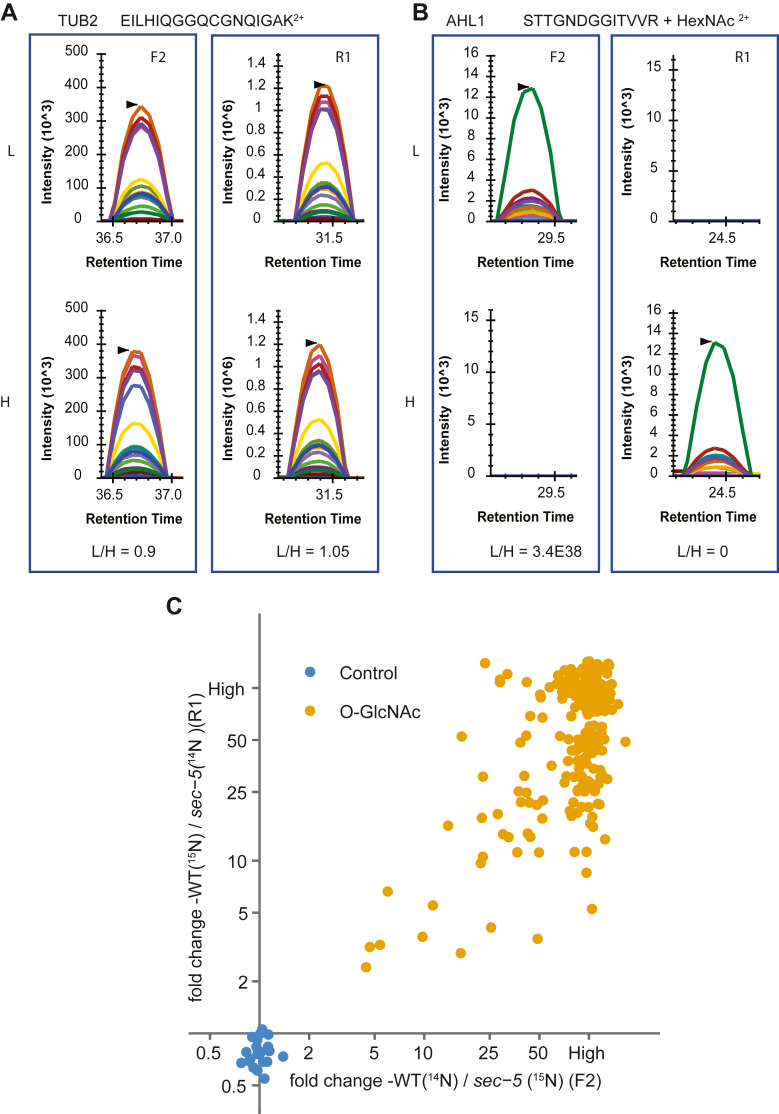
**Targeted quantification reveals reduced O-GlcNAcylation in *sec-5* mutant**. *A*, PRM data demonstrate equal abundance of TUB2 peptides between WT and *sec-5* mutants in F2 and R1 replicates. *B*, O-GlcNAc-modified peptides from AT-HOOK MOTIF NUCLEAR-LOCALIZED PROTEIN 1 (AHL1) protein show a significant reduction in *sec-5* mutant. Selected transitions were extracted for the ^14^N-labeled (L) and ^15^N-labeled (H) peptides. Area under the curve was used for ratio calculation. *C*, scatterplot displays ratios of O-GlcNAc-modified peptides and control peptides using SILIA–PRM. Quantification was performed in both F2 and R1 replicates, comparing WT *versus sec-5* mutants. PRM, parallel reaction monitoring; SILIA, stable isotope labeling in Arabidopsis.

Next, we performed targeted quantification using PRM in the F2 and R1 replicates. PRM overcomes the low identification issues in ^15^N-labeled samples as well as missing values between replicates (25). To select transitions, we chose y-type ion fragments because of their higher ion abundance compared with b-type ion fragments (22). We quantified 10 peptides with four to five transitions, whereas the rest of the peptides used ≥6 transitions to quantify the abundance. With these stringent criteria, we obtained high confidence in quantification. Consistent with MS1 quantification, PRM showed the control TUBULIN2 peptide having equal abundance between WT and *sec-5*. In contrast, O-GlcNAc peptide PRM shows a striking reduction in the *sec-5* mutant, as exemplified by a modified peptide from the AT-HOOK MOTIF NUCLEAR LOCALIZED PROTEIN 1 (AHL1) (Fig. 3*B*). In total, we quantified 172 O-linked peptides from 109 proteins using Skyline quantification (25, 31). Targeted quantification shows consistent reduction in the *sec-5* mutant, with only two O-GlcNAc-modified peptides showing about threefold median difference, whereas 170 show a significant difference (>5-fold median) (Fig. 3*C* and supplemental Data S1) in which 163 peptides show over 25-fold difference. In contrast, the N-linked peptides did not show consistent quantification difference across three replicates (supplemental Fig. S1).

Taken together, the quantitative results from these replicates on 184 peptides across 115 proteins using DDA and PRM suggest that SEC is a major contributor to O-GlcNAcylation in Arabidopsis.

### Large-Scale O-GlcNAc Peptide Identifications

The O-GlcNAcome database has comprehensively cataloged over 3000 and 1430 O-GlcNAc targets identified in humans and mice (15, 16). However, the database for O-GlcNAc targets in plants is still relatively limited. We previously reported 262 O-GlcNAc-modified proteins found in Arabidopsis inflorescence tissue. To further expand our knowledge of the O-GlcNAcome in plants, we performed additional LWAC enrichment with a series of comprehensive mass spectrometry (MS) runs, as detailed in the Experimental Procedures section.

In our measurement, we employed two acquisition approaches: the HCD method on Q-Exactive HF and the HCD/204 product-dependent EThcD analysis on Tribrid Eclipse. HCD is sensitive and effective in confidently identifying O-GlcNAc peptides, thanks to its short cycle time and efficient backbone fragmentation. However, it cannot assign modification sites because of the neutral loss of the very labile O-GlcNAc moiety from fragment ions. Conversely, ETD enables site assignments by producing c/z backbone fragment ions that retain the O-GlcNAc moiety, facilitating mass shifts for site localization (32). Nonetheless, ETD may lead to EtnoD, reducing the sensitivity of detecting O-GlcNAc-modified peptides (33).

To overcome these challenges, we utilized HCD/204pd-triggered EThcD, which provides a richer spectrum and yields more site assignments (34). Notably, fragments obtained from ETD activation retain the modification in EThcD, whereas those from HCD activation often do not for O-GlcNAc peptides. We therefore employed two methods, HCD-m or EThcD-m1, and EThcD-m2, for data searches. HCD-m or EThcD-m1 allows both loss and retention of the modification (S/T/N) on fragments (see Experimental Procedures section) for data acquired using HCD and EThcD; HCD-m considers b, y ions, whereas EThcD-m1 takes into account c, z, b, and y ions. Our in-house–installed software Protein Prospector treats them as separate options and reports the higher scoring result. EThcD-m2 is used for EThcD data and assumes all fragments are modified on S/T/N and considers c, z, b, and y ions.

To ensure the high-confidence identification of O-GlcNAc-modified proteins, we implemented several criteria for data filtering. These included examining if MS2 contains a 204-oxonium ion peak, or if peptides are identified by HCD and EThcD, or if modified peptides contain different miscleavages. We found several exceptions, such as the 204 ions being out of the scanning range in MS2 spectra for some peptides with a larger mass (over 2000). To address these cases, we manually examined their spectra and observed that the MS2 spectrum often accompanied the precursor-neutral loss of sugar (−203.08).

Our MS analysis of LWAC-enriched peptides, involving a total of 107 DDA measurements, yielded a total of 1721 O-GlcNAc peptides, corresponding to 1057 unique peptide sequences derived from 436 proteins (supplemental Data S3). When compared with our previous study that utilized LWAC-enriched samples from Arabidopsis inflorescence tissue (20), we discovered 227 new O-GlcNAc-modified proteins (supplemental Data S4). However, 53 proteins were not detected in this study, likely because of their specific expression in flower tissue.

**Fig. 4:**
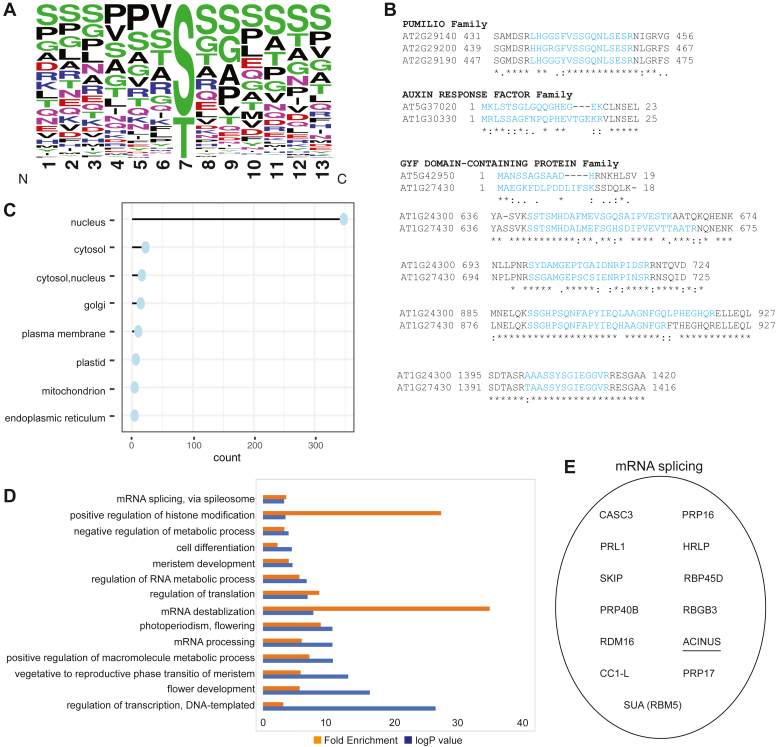
**Summary of identified O-GlcNAcylated proteins and peptides**. *A*, WebLogo motif representing the alignment of identified O-GlcNAc-modified sites. *B*, comparison of selected modified regions within the same modified family. *C*, SUBA analysis reveals predominant localization of O-GlcNAcylated acceptor substrate in the nucleus and cytosol. *D*, Gene Ontology (GO) analysis of *in vivo* detected O-GlcNAcylated proteins. *Yellow* indicates fold enrichment, and *blue* indicates log *p* value for biological processes on the *y*-axis. *E*, novel mRNA splicing factors are identified. Previously identified splicing factor ACINUS is underscored.

The data obtained from EThcD allowed us to successfully identify 596 O-GlcNAc modification sites in peptides (supplemental Data S3). Sequence analysis of O-GlcNAcylation sites did not reveal any consensus sequences, except for a slight preference for proline at the −2 or −3 N-terminal positions, and a slight preference for valine residues at −1 position to the modification site. Furthermore, serine residues were often observed at C-terminal positions to the modification sites (Fig. 4*A*). This finding is consistent with previous reports (20) as well as reports from animal studies (16, 35). These results align with the structure analysis of hOGT, indicating that it primarily binds to the peptide backbone of substrates and shows no clear specificity for the modifications of specific serine or threonine (36).

Given no clear consensus, to our surprise, we discovered O-GlcNAcylation occurring in various members of the same protein family (supplemental Data S4). For instance, we identified 20 modified family members belonging to the CCCH-type zinc finger family, 12 members from the AT HOOK MOTIF DNA-binding protein family, nine members from the ENTH/VHS protein family, nine members from TCP family transcription factor, eight members from the evolutionarily conserved C-terminal region proteins, eight members from the bHLH DNA-BINDING protein family, eight members from the homeodomain-containing proteins, six members from the PUMILIO FAMILY, five members from the OCTICOSAPEPTIDE/PHOX/BEM1P FAMILY PROTEIN, five members from the AUXIN RESPONSE FACTORs, and three members from the GYF domain–containing proteins.

We proceeded to examine the specific regions where these modifications occur within these proteins. Interestingly, despite the absence of the consensus sequences derived from the motif assay, we discovered that similar regions within the same protein family are subjected to modification (Fig. 4*B*). For example, in the mRNA binding protein family PUMILIO, we observed that PUMILIO 3 (AT2G29140), along with its homologs PUMILIO 1 (AT2G29200) and PUMILIO 2 (AT2G29190), are all modified at the same region, as indicated by the blue-colored modified peptides. Likewise, in the auxin transcription factors, we found that both AUXIN RESPONSE FACTOR 8 (ARF8, AT5G37020) and its homolog ARF6 undergo O-GlcNAc modification in their N-terminal regions. In addition, we observed that similar regions of the heavily modified GYF protein family (AT1G27430, AT1G24300) are also subject to modification (the shared identical peptides are not displayed here) (Fig. 4*B*). Based on these findings, we hypothesize that there may be adaptor proteins employed by SEC, similar to the proposed mechanism for hOGT, enabling the modification of different members from the same family (37). Further exploration of the O-GlcNAcome with even higher coverage will provide more opportunities for comparison and deeper insights into the OGT selectivity and specificity.

### SUBA and GO Analysis of O-GlcNAc Substrates

Subcellular localization analysis of the O-GlcNAc proteins using SUBA5.0 (26) revealed that the majority of the O-GlcNAc-modified proteins are predicted to be localized in the nucleus (Fig. 4*C* and supplemental Data S4). The next most abundant group of O-GlcNAc-modified proteins is predicted to be in the cytosol, whereas only a small number of proteins are predicted to be localized elsewhere. However, we noted that some SUBA predictions may not be accurate. For example, AT2G32080, PURIN-RICH ALPHA1, is predicted by SUBA to be localized to the Golgi, but TAIR predicts to be active in the nucleus. Considering its O-GlcNAc modification and its homologs’ involvement in transcriptional control and cytoplasmic RNA localization in animals (38), it is more likely to be cytosolic or nuclear.

GO analysis revealed a significant enrichment of crucial molecular functions associated with O-GlcNAc-modified proteins (Fig. 4*D*). These functions encompassed mRNA splicing, histone modification, cell differentiation, meristem development, translation, mRNA destabilization and processing, as well as transcription. Notably, a substantial portion of the identified O-GlcNAcome was found to be involved in RNA metabolism, histone modification, and translation, which mirrors observations in the human O-GlcNAcome, suggesting a conserved regulatory mechanism for O-GlcNAcylation in these processes.

mRNA splicing components are notably enriched in our findings (Fig. 4, *D* and *E*). Specifically, in addition to previously reported ACINUS, we identified 12 additional novel splicing factors, including CASC3, PRP16, PRL1, HRLP, SKIP, RBP45D, PRP40B, RBGB3, RDM16, CC1-L, PRP17, and SUA(RBM5). We previously showed that both SEC and SPY can affect splicing in Arabidopsis (39), and similar effects on splicing have also been observed when O-GlcNAc is perturbed in animals (40).

Furthermore, our analysis revealed a significant enrichment pointing to the central role of O-GlcNAc in signaling. For instance, we identified seven proteins harboring the WD40 (including NEDD1, VCS, TWD40–1, KTN80 Subunit 4, FY, PRP17, LUG) and 17 proteins with kinase domains as prominent targets of O-GlcNAcylation. These WD40 and kinase domains exhibit extensive interaction surfaces and are commonly associated with vital functions in signal transduction pathways.

### Crosstalk Between O-GlcNAcylation and O-fucosylation

**Fig. 5:**
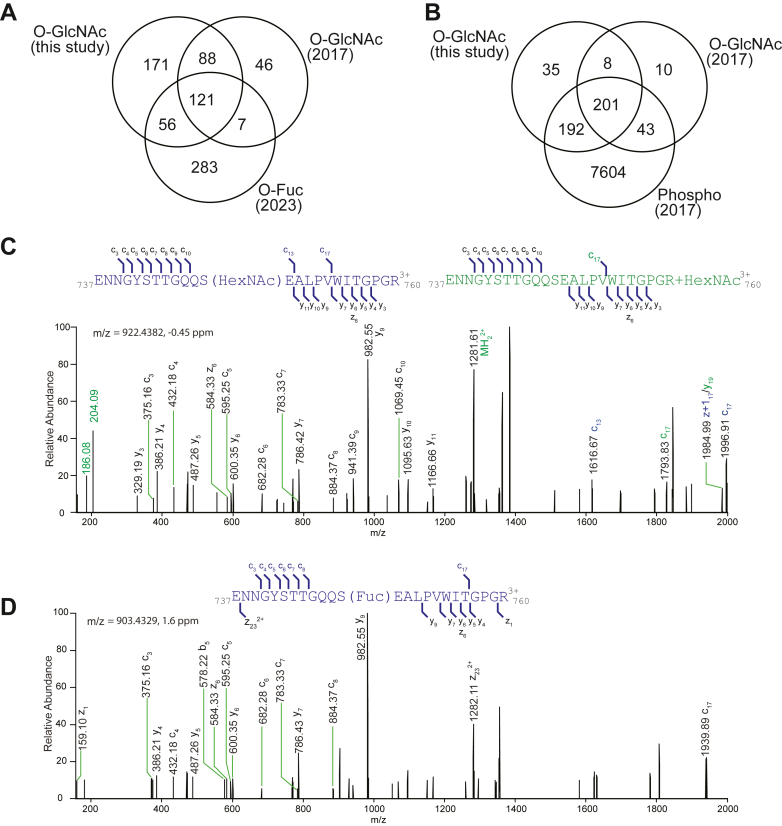
**Crosstalk between O-GlcNAcylation, O-fucosylation, and O-phosphorylation.***A*, a Venn diagram illustrates the intersection of O-GlcNAcylated proteins identified in this study, previously reported O-GlcNAcylated proteins, and O-fucosylated proteins. *B*, a Venn diagram demonstrates the overlap between O-GlcNAcylated proteins and O-phosphorylated proteins. *C* and *D*, EThcD spectra of *m/z* 922.4382 and *m/z* 903.4329 identify a peptide from KINASE-RELATED PROTEIN OF UNKNOWN FUNCTION (AT3G07660) being modified by O-GlcNAcylation or O-fucosylation at the same serine 748 site. *C*, EThcD spectra display fragments that contain modifications (highlighted in *blue*) or neutral loss (highlighted in *green*). EThcD, electron-transfer high-energy collision dissociation.

In our study, we identified a total of 436 O-GlcNAc targets, including 227 newly discovered targets, which complements the previously reported 262 targets and expands the total number of identified O-GlcNAc targets to 489 (Fig. 5*A*). Given both the known shared and distinct functions of SEC and SPY (41), we compared the O-GlcNAc and O-fucosylated datasets and were able to expand the shared targets from 128 (12) to 184, suggesting a strong potential crosstalk between these two PTMs, with over ⅓ of the targets overlapping between these two datasets.

Based on previous reported crosstalk between O-GlcNAcylation and phosphorylation (42), we further compared the O-GlcNAc datasets with phospho datasets (20). Of the 489 identified O-GlcNAc targets, a substantial 436 of them were also found to undergo phosphorylation (Fig. 5*B*). On the other hand, 17 proteins with kinase domains were identified as O-GlcNAc targets, so O-GlcNAcylation may affect the phosphorylation status by modulating the kinase activity. These observations underscore the intricate regulatory crosstalk.

Next, we explored whether the modifications occurred at the same sites by examining the data. Interestingly, we found certain proteins that were modified by both O-GlcNAcylation and O-fucosylation at precisely the same sites (supplemental Data S6). For instance, a peptide from KINASE-RELATED PROTEIN OF UNKNOWN FUNCTION was found to be modified at the serine 748 position by both O-GlcNAcylation and O-fucosylation (Fig. 5, *C* and *D*). To further map these sugar modifications, additional supporting data, particularly from ETD and EThcD analyses, would be valuable for in-depth investigation.

### Complexity With EThcD for Site Assignment

Despite significant efforts, assigning O-GlcNAc sites remains challenging because of two main factors. First, the modified peptide sequence, often found in intrinsic domains, tends to feature clusters of serine and threonine sites and requires significantly more fragments for unambiguous site assignment (15). Second, despite ETD, electron-transfer collision-induced dissociation, and EThcD showing promise for site assignment, their sensitivity is compromised by longer cycle times (32, 43). Furthermore, it is noteworthy that in HCD activation, the glycosidic bond of O-GlcNAc is more labile to cleavage compared with that of N-GlcNAc.

**Fig. 6:**
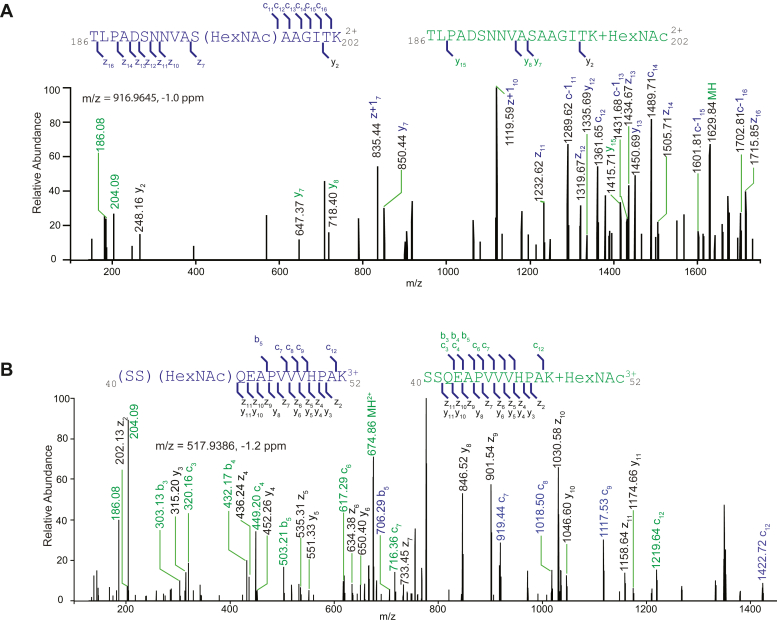
**Complexity of site assignment using EThcD**. *A*, fragments generated by ETD activation retain the modifications, whereas HCD fragments do not. The *m/z* 916.9645 identifies an O-GlcNAcylated peptide from ECT2 protein modified at serine 196. *B*, while some fragments are detected modified owing to ETD, but many more fragments unmodified owing to HCD activation. The *m/z* 517.9380 identified an O-GlcNAc-modified peptide from ECT5 protein modified at serine 40 or 41. The fragments retaining modifications are highlighted in *blue*, whereas the fragments that undergo neutral loss are highlighted in *green*. The ions supporting both annotations are colored *black*. ETD, electron-transfer dissociation; EThcD, electron-transfer high-energy collision dissociation; HCD, higher-energy collision dissociation.

To address these challenges, we employed EThcD-m1, which considers both the loss and retention of the modification on fragments. The Protein Prospector software treats these options separately and reports higher scoring results. In cases where EThcD-m1 scores are comparable, both possibilities are presented. If loss scores predominate, site information is excluded. To capture more site information, we also employed EThcD-m2, assuming all fragments are modified on S/T/N. As shown in Figure 6, *A* and *B*, when only assuming the fragments are modified, fragments (colored in *blue* and *black*) are assigned, but those with neutral loss (colored in *green*) remain unexplained. Figure 6*A* reveals a small number of unexplained fragments, whereas Figure 6*B* displays a more substantial quantity. These findings show similar trends as previously described (44) for the EThcD data. To further enhance site assignment accuracy and best benefit from the rich spectrum generated by EThcD, future algorithm advancements and improved scoring methods are necessary.

## Discussion

Recent discoveries have introduced several intriguing questions into our understanding of O-GlcNAcylation. Notably, the identification of an atypical OGT in wheat (14), the mild phenotype observed in the *sec-5* mutant (8), and the uncertain *in vivo* OGT activity of SPY collectively raise inquiries regarding the prominence of SEC as a primary contributor to O-GlcNAcylation. In addition, the substantial difference in expression levels between SEC (undetectable) and SPY (intensity-Based Absolute Quantitation = 22.47), akin to AT1G60140 (trehalose phosphate synthase) or AT3G51470 (protein phosphatase 2C) (45) and corroborated by our unpublished data encourages an exploration of the functional significance of SEC.

We first confirmed that SEC and SPY have redundant functions during embryogenesis and that O-glycosylation plays a crucial role in maintaining embryo cell viability. The failure to recover the *sec spy* double mutant despite extensive efforts underscores the functional overlap between these two genes.

The *in vitro* O-GlcNAcylation activity of SEC has been demonstrated, and transgenic DELLA proteins were found to exhibit reduced O-GlcNAc levels in the *sec* mutants (11, 46). In our comprehensive quantitative analysis, we identified a significant reduction in O-GlcNAc levels within *sec-5* mutants. Utilizing both DDA and targeted quantification through PRM, we established that SEC predominantly drives O-GlcNAcylation in plants. Despite observing comparatively lower O-GlcNAc enrichment in Arabidopsis seedlings compared with previous flower tissues, our SILIA labeling approach at the seedling stage enabled precise quantification, confirming the major contribution of SEC to O-GlcNAcylation *in vivo*.

Detecting O-GlcNAc modification is challenged by its substoichiometry. For instance, in unenriched samples, only 126 O-GlcNAc-modified peptides were detected among 14 million spectra (47). To expand the plant O-GlcNAc repertoire, we combined LWAC enrichment with extensive high-pH reverse HPLC fractionations, which enabled the identification of 227 new targets, expanding our O-GlcNAcome database to encompass a total of 489 targets.

Alternative approaches using click chemistry have been employed to detect O-GlcNAc targets in Arabidopsis seedlings (18). By supplementing GalNAz and GlcNAz, metabolic glycan labeling was induced in Arabidopsis seedlings. Given GlcNAz’s toxicity, the enrichment focused on GalNAz-labeled samples. Subsequent click chemistry and enrichment through streptavidin beads enabled the profiling of 645 O-GlcNAc targets. Our comparative analysis revealed only 68 overlaps in seedling datasets and 75 in overall datasets. Notably, click chemistry enriches targets predominantly associated with cellular amide metabolic processes (18).

Given the observed redundant functions of O-GlcNAc and O-fucosylation during embryo stages, and the mild phenotype of SPY and SEC postembryo development, the data strongly suggest an extensive crosstalk between O-GlcNAc and O-fucosylation, implying the potential targeting of the same substrates. Our comprehensive profiling of O-GlcNAc datasets has significantly expanded the number of overlapping substrates to 189, encompassing almost one-third of the detected targets. Remarkably, aside from the previously documented contrasting effects on the DELLA group (11, 46), recent findings illustrate that SEC and SPY collaboratively regulate style development through the bHLH transcription factor SPATULA (48). These new insights uncover novel roles for O-glycosylation and synergistic effects of these two sugar modifications. Notably, the site-level overlap is still significantly lacking, potentially hindering further scrutinizing of the crosslink mechanism. While immunoprecipitating the individual target proteins followed with MS analysis will enhance the site overlapping (48), a systems-level optimization, including modified peptide enrichment methods along with the EThcD site assignment algorithm, will be instrumental in advancing this exciting “sweet” field.

## Data availability

The MS proteomics data have been deposited to the ProteomeXchange Consortium *via* the PRIDE partner repository with the dataset identifiers PXD044668, PXD044832, PXD044831, PXD044675, and PXD044701. All other data are available from the corresponding author on reasonable request. The spectra for the glycosylated peptide identification can be viewed using MSviewer (49), with search key iwfsqsypya for N14 HCD spectra, rulbqlodsc for N14 EThcD spectra, and qnjmnfmczr for N15 HCD spectra.

## Supplemental data

This article contains supplemental data.

## Conflict of interest

The authors declare no competing interests.
