# Supplementary figures and images for: SECRET AGENT O-GlcNAcylates Hundreds of Proteins Involved in Diverse Cellular Processes in Arabidopsis
Source: Mol Cell Proteomics. 2024 Feb 8;23(4):100732. doi: 10.1016/j.mcpro.2024.100732 (PMC10979276; doi:10.1016/j.mcpro.2024.100732)

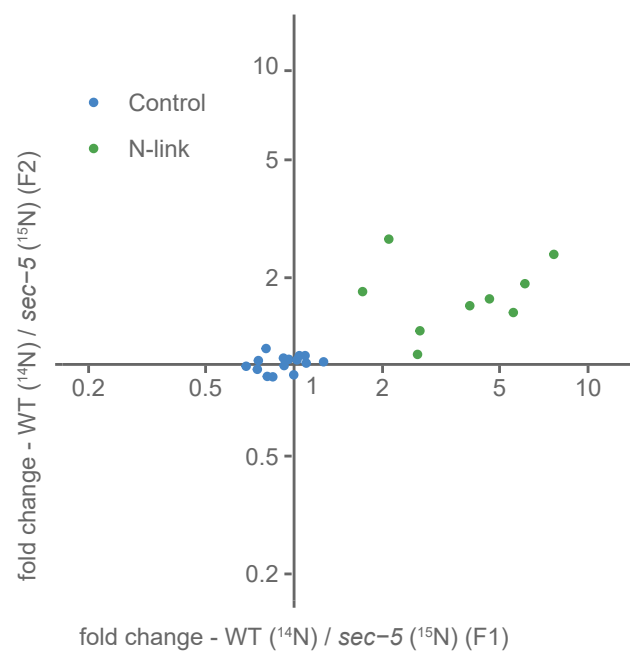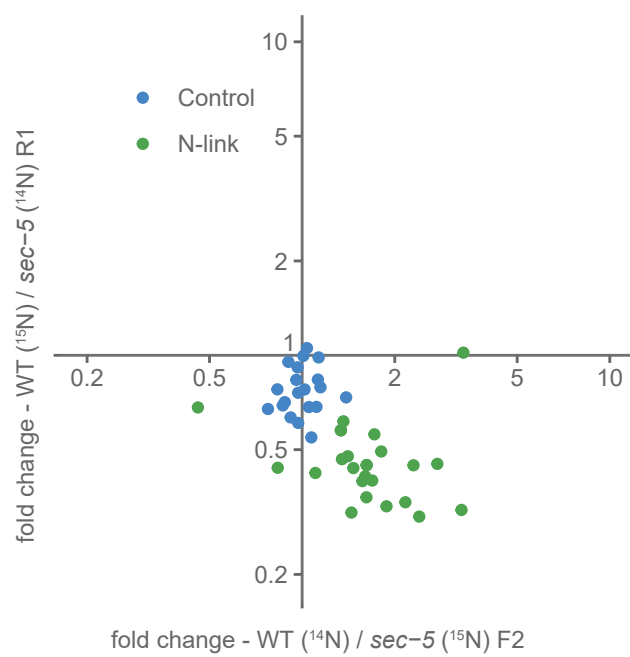

Supplement: Supplemental Fig. 1 [file mmc9.pdf]

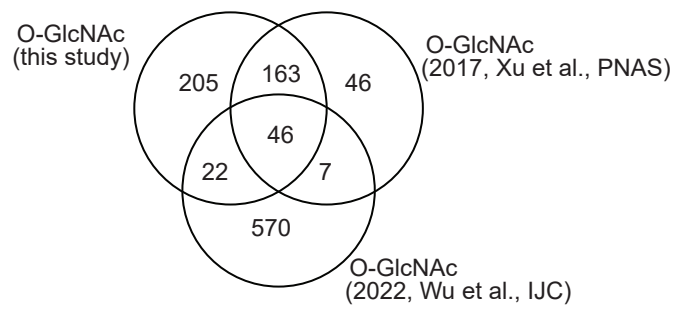

Supplement: Supplemental Fig. 2 [file mmc10.pdf]
